# Oxytocin release patterns and effects, with a focus on touch, social interaction, and closeness

**DOI:** 10.3389/fpsyg.2026.1771799

**Published:** 2026-06-24

**Authors:** Kerstin Uvnäs-Moberg, Maria Petersson

**Affiliations:** 1 Department of Animal Environment and Health, Section of Anthrozoology and Applied Ethology, Swedish University of Agricultural Sciences, Skara, Sweden; 2 Department of Endocrinology, Karolinska University Hospital and Department of Molecular Medicine and Surgery, Karolinska Institutet, Solna, Sweden

**Keywords:** anti-stress effects, birth, breastfeeding, closeness, growth, health, kangaroo treatment, massage

## Abstract

Clinical evidence indicates that tactile stimulation of the skin, such as stroking or gentle touch, elicits a range of beneficial physiological and psychological responses, such as increased social interaction, wellbeing, calm, restorative and growth promoting effects, as well as reduced fear, pain, and stress levels. These effects are mediated by activation of cutaneous sensory nerves, which relay signals to central regulatory systems. During this process, several neuropeptides and hormones are released, one of which is oxytocin produced in the supraoptic (SON) and paraventricular (PVN) nuclei of the hypothalamus. Oxytocin in turn stimulates social interaction, induces wellbeing and calm, and promotes growth and restorative processes in part via vagal activation of the endocrine system of the gastrointestinal tract and by inhibiting the activity within the hypothalamic pituitary adrenal (HPA)-axis and the sympathetic nervous system. It is proposed that cutaneous nerves are typically activated during social interaction through a two-step process. Gentle touch activates a two-neuron pathway involving tachykinin 1-neurons originating from the periaqueductal gray (PAG) and thereafter projecting to the SON and PVN. This activation triggers the release of oxytocin from neurons in the SON and PVN, which by activating oxytocin receptors promotes social interaction and rewarding mechanisms. Subsequent behaviors such as holding and caressing, trigger a second pool of cutaneous afferents, that respond to higher mechanical pressure than those triggered by gentle touch. We propose that these fibers are associated with oxytocin linked calming effects and with enhanced metabolic, restorative and growth promoting benefits, e.g., via vagal activation of the endocrine system of the gastrointestinal tract. In addition, anxiety, stress, pain and inflammation levels are decreased via oxytocinergic mechanisms in the brain. It is also possible that oxytocin released in response to cutaneous afferents exerts long-term effects by increasing oxytocin production and enhancing the function of oxytocin receptors. These two types of oxytocin-associated effect patterns may be activated, to varying degrees, in clinical practices involving skin-to-skin contact between individuals in certain therapeutic or caregiving contexts, such as skin-to-skin contact between infant and parent after birth or in the more long-term perspective during kangaroo care. Consequently, lack of social contact and closeness leads to reduced capacity for social interaction, increased stress levels, impaired health and even retarded growth at young age. Birth and breast-feeding, but not touch or closeness, are associated with a pulsatile release of oxytocin into the circulation. In addition, oxytocin linked pathways in the brain will be activated in response to birth and breastfeeding just as in response to touch and closeness. The release and effects of oxytocin will depend on the intensity and location of the cutaneous stimulation and of the oxytocin release pattern induced. Given the potential for different effects in response to distinct types of cutaneous stimulation manual therapies could be designed for targeted effects, such as reducing stress, relieving pain, boosting calm, or even promoting growth in early life. The aim of this narrative review was to explore the potential roles of central oxytocin, including its release and associated effects, in response to different types of sensory stimulation. In addition, we examine how oxytocin may contribute to social interaction and to clinical practices involving touch or close physical contact.

## 1 Oxytocin

### 1.1 The oxytocin system

Oxytocin is produced in neurons within the supraoptic (SON) and paraventricular (PVN) nuclei of the hypothalamus. Magnocellular neurons project to the posterior pituitary, wherefrom oxytocin is released into the bloodstream, e.g., during labor and breastfeeding. In addition, a specific type of oxytocin neurons, the parvocellular neurons, originating in the PVN as well as axon collaterals from the magnocellular neurons from the SON and PVN project to multiple areas within the central nervous system, where they participate in the control of various physiological and behavioral functions (Buijs, 1983; Burbach and Young, 2006; Sofroniew, 1983; Wang et al., 2022).

Although oxytocin was originally thought to be a female hormone, it is now well established that oxytocin release and effects are exerted irrespective of sex—from infancy to old age.

The areas receiving oxytocinergic innervation include, but are not restricted to, the frontal cortex, amygdala, hippocampus, bed nucleus of the stria terminalis (BNST), locus coeruleus (LC), the rostral ventrolateral medulla (RVLM), PAG, striatum, nucleus accumbens (NA), the raphe nuclei, the pineal gland, the sympathetic ganglia, the dorsal vagal motor nucleus (DMX), the nucleus of the solitary tract (NTS) in the brain stem, pain mediating neurons in the spinal cord and parasympathetic ganglia in the lumbosacral region (Buijs, 1983; Burbach and Young, 2006; Sofroniew, 1983; Stoop et al., 2015; Wang et al., 2022). The list of areas within the brain that receives oxytocinergic innervation is constantly growing reflecting the development of new and more sensitive techniques for demonstration of the projections from the oxytocin neurons (Wang et al., 2022). New techniques also allow studies of the release of oxytocin from separate neuronal bundles in the brain, and how different oxytocin projections may be sequentially activated (Neumann and Landgraf, 2019; Qian et al., 2023). Oxytocin released within the central nervous system does not always act as a classical neurotransmitter via synaptic transmission, since it also has the capacity to exert paracrine actions by reaching oxytocin receptors by diffusion (Ludwig and Leng, 2006; Del-Bel and De-Miguel, 2018; Knobloch and Grinevich, 2014).

### 1.2 Effects of oxytocin

Oxytocin was originally known to facilitate milk ejection and to promote uterine contractions via hormonal actions. However, oxytocin released from oxytocinergic neurons in the brain participate in the regulation of a multitude of behavioral and physiological effects. These effects of oxytocin include an increased social motivation and competence for social interaction. It also promotes growth and tissue repair, including cell regeneration and restoration of atrophied tissues, as well as activation of the vagal/parasympathetic pathways with activation of the endocrine system of the gastrointestinal tract. Oxytocin is also linked to reduction of fear, stress, pain, and inflammation. These effects are in part exerted via decreased activity of the amygdala, of the HPA-axis and of the sympathetic nervous system (Chen et al., 2009; Neumann and Landgraf, 2019; Uvnäs Moberg et al., 2024).

### 1.3 Stimulation of reproduction, restoration, and growth

The ability of oxytocin to activate the endocrine system of the gastrointestinal tract plays a very important role in processes related to restoration, growth and reproduction. As mentioned above, oxytocin neurons project to the DMX and the NTS where efferent vagal nerve fibers, that stimulate gastrointestinal function including gastrointestinal peptides and hormones, originate (Buijs and Van Heerikhuize, 1982; Uvnäs Moberg et al., 2024).

### 1.4 The endocrine system of the gastrointestinal tract

The gastrointestinal tract contains more than a hundred hormones and peptides, which exert multiple effects on digestion, metabolism and growth. The release and activity of many of these hormones are regulated by the vagal nerve which may induce multiple both inhibitory and stimulatory effects (Uvnäs Moberg, 2024; Waise et al., 2018).

### 1.5 The inhibitory function of somatostatin

Somatostatin is a significant gastrointestinal hormone that was originally shown to inhibit the secretion of growth hormone from the pituitary gland. In addition, it is produced and released by a widespread network of somatostatin-secreting cells located in both the gastrointestinal tract and the endocrine pancreas. Somatostatin exerts a general inhibitory effect on the function of the gastrointestinal tract including the release and effects of multiple hormones of the gut and the pancreas. In this way somatostatin acts like a general brake on the function of the endocrine system of the gastrointestinal tract. Consequently, it inhibits digestive and anabolic functions, restorative processes and growth (Uvnäs Moberg, 2024). Sick infants and infants that are small for date have higher somatostatin levels than those who are healthy (Marchini et al., 1988). Thus, somatostatin inhibits growth in two ways, by inhibiting the release of growth hormone from the pituitary and by inhibiting the function of the gastrointestinal tract including the endocrine system of the gastrointestinal tract (Van Op den Bosch et al., 2009; Uvnäs Moberg, 2024).

### 1.6 Oxytocinergic nerves may inhibit the release of somatostatin via a vagal mechanism

Central oxytocin may interact with somatostatinergic function via vagal pathways, and experimental findings suggest that oxytocin can modulate somatostatin release. Several studies report an inverse relationship between oxytocin and somatostatin levels, with higher oxytocin concentrations being associated with lower somatostatin levels in both humans and animal models. Such inhibitory modulation is thought to be mediated, at least in part, through an oxytocin mediated enhanced vagal signaling to the gastrointestinal tract. These effects could influence gastrointestinal endocrine function, including processes related to digestion, metabolism, and anabolic activity.

These mechanisms may be particularly important early in life, when gastrointestinal hormones, including insulin, partly compensate for the relatively low activity of the growth hormone axis during this developmental period (Silber et al., 1991; Benyi and Sävendahl, 2017; Uvnäs Moberg, 2024).

### 1.7 Inhibition of stress, fear, and pain—Evidence from experimental studies

Oxytocinergic nerves counteract the activity of the stress axis at multiple sites. Both oxytocin and corticotrophin releasing factor (CRF) are produced within the PVN. Oxytocin released within the PVN decreases the release of CRF from neurons in the PVN via activation of GABAergic interneurons acting on GABA A receptors (Takahashi, 2021). In addition, parvocellular oxytocin neurons and oxytocin released from axon collaterals of the magnocellular neurons projecting to the amygdala decrease the levels of fear, and other fibers project to the median eminence to decrease the release of adrenocorticotropic hormone (ACTH) from the anterior pituitary. Oxytocinergic neurons also counteract sympathetic nervous activity indirectly through the inhibition of CRF release but also directly by actions in the LC, the RVLM, and the presynaptic sympathetic ganglia (Buijs and Van Heerikhuize, 1982; Gryksa et al., 2025; Uvnäs Moberg et al., 2024). Oxytocin decreases blood pressure by effects within the RVLM. However, oxytocin also influences cardiovascular function by actions in the DMX, where it reduces heart rate and contributes to heart rate variability (HRV) (Dellacqua et al., 1985; Higa et al., 2002; Wang et al., 2022).

### 1.8 The calm and connection system

When the oxytocinergic system is significantly activated, social interaction and calmness will prevail, and growth activities are promoted while the activity of the stress systems is reduced. These combined effects of oxytocin: the vagally mediated growth promoting effects, the anti-stress effects and the facilitating effects on social interaction together form *the calm and connection system*. We propose that promoting restoration, growth, and reproduction constitutes a fundamental and archaic aspect of the oxytocin effect pattern. As these anabolic processes demand substantial energy, oxytocinergic fibers facilitate optimal energy allocation by suppressing other energy-intensive physiological activities, such as stress responses and inflammation (Uvnäs Moberg, 2024).

### 1.9 Differential effect pattern in response to different doses of oxytocin

When administered to animals, the behavioral and physiological effects of oxytocin can be categorized based on the dosage. Intracerebroventricular (icv) injection of oxytocin in nanogram amounts (low dose) to rats enhances locomotor activity in the central parts of the arena during the open-field test, indicating anxiolytic-like and explorative behaviors (Uvnäs-Moberg et al., 1992). This oxytocin-induced behavior, characterized by activity and curiosity is necessary for initiation of social interaction. In addition, this dose of oxytocin has been shown to promote social approach behavior and to facilitate social recognition in rodents (Lukas et al., 2011). By contrast, oxytocin administered icv in microgram amounts (high dose) reduces locomotor behavior and moves it into to the peripheral parts of the arena in the open field test. This behavioral effect pattern reflects increased calmness and a sedative effect (Uvnäs-Moberg et al., 1994).

In addition, the higher dose of oxytocin has been shown to support physiological processes related to digestion, anabolism, tissue restoration and growth, and to exert antistress, analgesic and anti-inflammatory effects contributing to homeostasis and recovery. Furthermore, in animal studies, the function of certain populations of central alpha-2 adrenoceptors including those linked to the noradrenergic activity in the LC is enhanced (Petersson et al., 1998). This effect may be an important factor underlying the growth promoting, restorative, analgesic as well as the antistress effects induced by the high dose oxytocin. Additionally, oxytocin in the high dose range increases opioidergic function thus contributing to the pain-relieving effects (Petersson et al., 1996). In addition, the effects induced by the “high” dose of oxytocin administered repeatedly become sustained for days or weeks after treatment cessation (Uvnäs-Moberg et al., 2015; Uvnäs Moberg, 2024).

Given the differences in oxytocin dosage required to elicit activating vs. calming effects, we have hypothesized that oxytocin may partially exert its actions via distinct receptors, potentially following degradation into active metabolites (Uvnäs-Moberg et al., 2019a,b).

## 2 Oxytocin release and effects in response to activation of sensory nerves

One of the aims of this article is to explore potential oxytocin release patterns and their associated effects when activated by different types of sensory nerves, particularly those originating in the skin. For comparative purposes, fundamental data on oxytocin secretion during classical physiological processes such as childbirth and lactation will be provided (Table 1).

**Table 1:** The effects of light or deep touch and breastfeeding.

Type of tactile stimulation	Primary Nerve Fibers/Receptors	Peptides/Transmitters released	Effects
Light (gentle) touch	CT-fibers Mechanoreceptors	xytocin Dopamine	Social interaction, wellbeing Anxiety↓
Firm touch	A-beta-fibers Mechanoreceptors	Oxytocin Dopamine Opioids Noradrenaline ↓	Calm, growth, reproduction, pain↓, blood pressure↓ Cortisol↓↓
Breastfeeding	A-beta-fibers Mechanoreceptors	Pulsatile oxytocin Dopamine Opioids Noradrenaline↓	Calm, growth, bonding, pain↓ blood pressure↓ heart rate↓ Cortisol ↓↓

### 2.1 Birth and the Fergusson reflex

During birth, oxytocin is released into the circulation in a pulsatile manner. This release is initiated by the activation of parasympathetic nervous afferents—a process known as the Fergusson reflex—which occurs when the fetal head applies pressure to the cervix and vaginal wall. The parasympathetic afferents subsequently stimulate noradrenergic neurons in the NTS, which then activate oxytocin-producing neurons within the PVN and SON. These neurons project to the posterior pituitary, resulting in oxytocin secretion into systemic circulation. Additionally, oxytocin is released within the brain from axon collaterals of magnocellular neurons targeting the posterior pituitary, and from parvocellular neurons from the PVN. These mechanisms not only facilitate social behaviors but also reduce fear, stress, and pain levels and they enhance the potential for restoration and growth (Ferguson, 1941; Knobloch et al., 2012; Uvnäs-Moberg et al., 2019a,b).

### 2.2 Suckling

During breastfeeding, the pressure generated by the infant's suckling activates specialized cutaneous sensory nerves originating in the nipple. Suckling elicits a pulsatile release of oxytocin into the circulation from magnocellular neurons located in the SON and PVN nuclei, which project to the posterior pituitary. *The higher the suckling induced pressure on the nipple, the more oxytocin is released, and the more milk is ejected*. These magnocellular neurons possess axon collaterals that extend to regions such as the median eminence, amygdala, and prefrontal cortex. Furthermore, parvocellular oxytocinergic neurons from the PVN project to several key regulatory areas within the brain, as well as to vagal nuclei in the brainstem. Oxytocin released in these regions facilitates social interaction, enhances feelings of wellbeing and calm, and reduces fear, stress, and pain, thereby adapting maternal physiology and behavior to motherhood. Additionally, oxytocin supports the function of the gastrointestinal endocrine system, optimizing digestive and anabolic processes vital for milk production and overall growth during lactation (Uvnäs Moberg, 2024).

### 2.3 Gastrointestinal vagal afferents

Oxytocin release is also influenced by sensory information from the gastrointestinal tract. The vagal afferents originating from the gastrointestinal tract stimulate oxytocin release in response to various stimuli, including mechanical pressure on the gastrointestinal wall and the presence of specific food constituents and even lactobacilli within the gastrointestinal lumen (Weber et al., 2024). These afferent vagal signals are transmitted to the SON and PVN via noradrenergic nerves arising from the NTS. By promoting the release of oxytocin, the presence of food in the gastrointestinal tract facilitates the release of oxytocin, e.g., during lactation (Lindén A, 1990; Eriksson et al., 1996; Uvnäs Moberg, 2024).

#### 2.3.1 Possible inhibition of somatostatin release

Evidence suggests that oxytocin may interact with somatostatinergic pathways during breastfeeding, Oxytocin increases parasympathetic activity and circulating oxytocin levels have been reported to vary inversely with somatostatin concentrations around parturition and during suckling-related stimulation. Moreover, oxytocin-associated increases during breastfeeding have been linked to vagally mediated reductions in somatostatin activity. These observations, together with findings showing that maternal oxytocin levels correlate with both infant birth weight and milk production, support that oxytocin contributes to activation of the gastrointestinal endocrine system during breastfeeding (Silber et al., 1991; Uvnäs Moberg, 2024).

### 2.4 Cutaneous sensory nerves

#### 2.4.1 The skin is a sensory organ

The skin serves not only as a protective barrier against environmental factors but also functions as the body's largest sensory organ. It contains numerous receptors that detect various stimuli and relay this information to the central nervous system through cutaneous sensory nerves. Both myelinated and unmyelinated fibers are involved in this process. Myelinated fibers quickly transmit sensory signals to the sensory cortex, helping identify where the stimulus is located. In contrast, unmyelinated C-fibers relay information at a slower pace, with this input being processed in the lower limbic regions of the brain (Glatte et al., 2019).

#### 2.4.2 Noxious and non-noxious sensory innervation of the skin

Certain cutaneous receptors respond to painful stimuli and tissue damage, while others are sensitive to gentler sensations like touch, stroking, light pressure, and warmth. These forms of stimulation are typically classified as noxious (harmful) and non-noxious (harmless), with both types potentially involving unmyelinated or myelinated sensory nerve fibers. Activation of noxious nerves is associated with a direct stimulation of the CRF-system within the PVN, which promotes the activity of the HPA-axis and of the sympathetic nervous system. Such stimulation also gives rise to sensations of fear, pain, and stress, which in turn leads to further activation of both the HPA-axis and the sympathetic nervous system. By contrast, stimulation of non-noxious or innocuous sensory nerves induces calming and restorative effects through oxytocin release from parvocellular oxytocinergic neurons in the PVN. Non-noxious stimulation activates the parasympathetic nervous system, indirectly via oxytocinergic projections to the DMX, as well as directly through sensory input at the brain stem level. Oxytocin-mediated behavioral and physiological changes resulting from non-noxious sensory stimulation align with the calm and connection response. Further discussion of these mechanisms will be provided in subsequent sections (Sato et al., 1997; Takahashi, 2021; Uvnäs Moberg, 2024).

### 2.5 Oxytocin release in response to cutaneous sensory stimulation

The choice of method for analyzing oxytocin levels in blood significantly affects the results. For example, enzyme-linked immunoassay (ELISA) and radioimmunoassay (RIA) can yield very different outcomes. Generally, RIA is considered more reliable than ELISA, as ELISA often reports higher baseline levels of oxytocin and may not detect smaller changes resulting from physiological stimulation. This is partly because RIA uses high-affinity antibodies and a radioisotope-based detection system, which minimizes interference and cross-reactivity, making it more specific and sensitive compared to the enzyme-linked colorimetric reactions in ELISA. However, some newer ELISA techniques produce lower oxytocin levels that are closer to those measured by RIA. Future measurements of oxytocin are likely to employ HPLC followed by quantification using mass spectrometry. Nevertheless, this methodology requires further validation, as recent findings have diverged from previously established data regarding oxytocin concentrations (McCullough et al., 2013). Given the variability introduced by differing analytical approaches, comparing oxytocin levels across studies employing distinct methodologies is not advisable (Uvnäs Moberg et al., 2020).

#### 2.5.1 Link between oxytocin release and the skin

The relationship between skin stimulation and oxytocin release has been established through animal studies. Research indicates that low-intensity electrical stimulation of cutaneous nerves results in oxytocin being released both into the brain and systemic circulation, with increased stimulation leading to greater oxytocin output (Stock and Uvnäs-Moberg, 1988). Additionally, tactile interactions such as touch and massage-like stroking have been shown to provoke oxytocin release in rats (Kurosawa et al., 1995; Holst et al., 2005). Touch has also been demonstrated to enhance the synthesis of oxytocin mRNA in the PVN and SON regions. The neurogenic pathway involved is likely composed of two neurons, with the second neuron projecting either directly or via axon collaterals to oxytocin-producing cells in these regions (Takahashi, 2021; Yu et al., 2022a,b; Tang et al., 2020).

#### 2.5.2 Patterns of oxytocin release

Both labor and suckling are associated with a pulsatile release of oxytocin into the circulation, as previously described. However, non-noxious skin stimulation does not produce this effect. The differing plasma patterns of oxytocin underscore that distinct neurogenic pathways and cellular mechanisms underlie these different release processes.

The release of oxytocin into the circulation following non-noxious stimulation is contingent upon the intensity of the stimulus and may vary between species. For example, gentle stroking of a cow's abdomen does not increase circulating oxytocin levels, despite the presence of oxytocin-mediated behavioral and physiological effects (Wredle and Svennersten-Sjaunja, 2022). In contrast, in dogs separated from their owners, plasma oxytocin levels increased when the dog first saw the owner re-enter the room, and further elevations were observed during and after physical contact between the dog and its owner (Rehn et al., 2014).

However, the absence of elevated plasma levels of oxytocin should not be interpreted as inactivity of the oxytocin system. Oxytocin may still be released centrally from parvocellular neurons in the PVN, and such central release is not necessarily reflected in peripheral blood concentrations. Studies by Kendrick and colleagues demonstrate that oxytocin can be released simultaneously in the brain and into the circulation, but also independently, indicating that central and peripheral oxytocin release may follow partially distinct regulatory mechanisms (Kendrick, 2000; Wredle and Svennersten-Sjaunja, 2022).

#### 2.5.3 Effects of oxytocin in response to stimulation of cutaneous sensory nerves

Stimulation of cutaneous sensory nerves has been linked to various oxytocinergic effects, both directly and indirectly, including enhanced social interaction, reduced levels of fear, stress, and pain, as well as improved gastrointestinal endocrine function that supports growth (Uvnäs Moberg, 2024).

Gentle stroking of the abdomen in cows has been linked to enhanced social interaction as well as to reductions in heart rate and cortisol levels. Notably, this intervention does not correspond with a decrease in ACTH levels, which is secreted by the anterior pituitary in response to elevated stress and CRF. These findings indicate that subtle tactile stimulation may act at a lower functional level within the hypothalamus compared to stronger sensory inputs. This type of stimulation may likely involve parvocellular oxytocin neurons in the PVN to a greater extent and can be triggered by a shift in autonomic nervous activity from sympathetic to parasympathetic dominance, affecting both the sympathetic preganglionic neurons and the DMX. This autonomic shift reduces the adrenal cortex's responsiveness to ACTH, thereby lowering cortisol levels independently of ACTH concentrations in the blood. A similar pattern of decreased cortisol—without reduced ACTH levels —is observed in breastfeeding mothers after a period of skin-to-skin contact with their babies (Handlin et al., 2009; Wredle and Svennersten-Sjaunja, 2022; Uvnäs-Moberg et al., 2015).

In contrast a massage like treatment in rats consisting of firm stroking of the ventral side (20 strokes per minute for 5 min) is associated with a rise of oxytocin levels, sedation, lowering of cortisol levels, blood pressure and heart rate as well as increased nociceptive thresholds. In addition, levels of gastrointestinal hormones including insulin are increased via activation of vagal mechanisms. This pattern of effects is comparable to that observed following administration of a high dose of oxytocin to rats, which has also been linked to increased activity of alpha-2 adrenoceptors (Holst et al., 2005; Petersson et al., 1998; Uvnäs Moberg, 2024).

## 3 Social interaction consists of two sequential phases, each potentially characterized by different oxytocin release patterns triggered by sensory nerve stimulation from the skin

Social interaction involves several behavioral components and physiological adaptations that typically occur in sequence. The initial phase often includes subtle, gentle touch, which serves to initiate contact and orient individuals toward one another. Within the human literature, this type of gentle touch corresponds closely to the Affective Touch framework proposed by McGlone and colleagues (McGlone et al., 2014). Affective touch refers to slow, light stroking of hairy skin at velocities of approximately 1–10 cm/s, optimally around 3 cm/s, which preferentially activates unmyelinated C-tactile (CT) afferents. Activation of CT afferents is reliably associated with subjective pleasantness and engages insular cortex regions involved in emotional processing (Löken et al., 2009; Schirmer et al., 2023).

Importantly, affective touch has been linked to oxytocin-related processes. Experimental studies show that oxytocin can enhance the perceived pleasantness of CT-optimal touch and modulate associated cortical responses (Chen et al., 2020), and that pleasant touch may itself be associated with increases in salivary oxytocin (Portnova et al., 2020). These findings provide a well-defined neurophysiological and psychophysical basis for understanding how gentle touch may initiate social approach, reduce anxiety, and promote wellbeing.

Animal studies support a similar mechanism. In mice, pleasant touch activates a two-neuron pathway involving tachykinin-1 neurons in the PAG projecting to oxytocin neurons in the PVN. Oxytocin release subsequently facilitates social interaction, activates dopaminergic reward pathways, and induces positive affective valence toward both the interaction partner and the context (Yu et al., 2022a,b). These findings align well with the affective touch framework and suggest that CT-optimal touch in humans may engage comparable oxytocinergic circuits.

Subsequent phases of social interaction often involve firmer or more static tactile engagement, such as holding or sustained pressure. This type of stimulation activates a different population of sensory afferents—likely including deeper mechanoreceptors—and engages distinct oxytocinergic pathways within the PVN. These pathways are associated with more pronounced antistress effects, reductions in fear and pain, and enhanced anabolic and growth-promoting processes, collectively contributing to the calm-and-connection response (Holst et al., 2005; Olausson et al., 2010; Uvnäs Moberg, 2024).

Thus, the differing effects of gentle vs. firmer tactile stimulation—ranging from social approach and pleasantness to profound antistress and growth-promoting outcomes—may reflect the activation of distinct sensory pathways and partially separate oxytocinergic circuits. This interpretation is consistent with behavioral and physiological findings in rodents showing that low vs. high doses of oxytocin produce different profiles of social, emotional, and metabolic effects (Uvnäs-Moberg et al., 1992, 1994; Uvnäs Moberg, 2024).

### 3.1 Gentle touch in humans

In humans, gentle touch is defined by light stroking of the skin at a velocity of approximately 3 cm/s, which stimulates a particular subset of C-fibers known as Ct-fibers. Individuals who receive gentle touch typically orient themselves toward the individual who is the source of touch and initiate social interaction. Gentle touch is associated with a sense of wellbeing and activation of insular cortex regions associated with positive emotions (Olausson et al., 2010). Based on the data from animal studies described above, it may be inferred that “gentle touch” in humans activates tachykinin −1 neurons in the PAG, which in turn activates oxytocinergic neurons in the PVN, which subsequently promote social interaction, decrease anxiety levels, and enhances dopaminergic functions and wellbeing. Furthermore, a positive perception of not only the individual providing the gentle touch but also of the location where touch was received, might be induced (Ellingsen et al., 2015; Pawling et al., 2017; Yu et al., 2022a,b; Walker et al., 2017).

Human social interaction may subsequently involve more robust sensory stimulation, such as that produced by holding. This type of physical contact may promote the calming, sedative, and stress-relieving effects associated with oxytocin release.

Some earlier observations regarding the positive effects of social interaction may be interpreted in light of oxytocin-linked mechanisms activated by tactile stimulation. For example, the reduced social competence and lower anxiety levels observed in Harry Harlow's monkeys reared by a surrogate mother constructed from steel wire, as compared to those raised by the mother or by fur-covered surrogate mothers, may, at least in part, be attributed to insufficient stimulation of cutaneous sensory nerves linked to oxytocin release in the group residing with the wire surrogate mother (Harlow, 1958).

The comforting effects of physical holding were first recognized and articulated by John Bowlby (Bowlby, 1969). When a mother picks up a crying infant and holds the child close to her chest, this physical embrace rapidly alleviates distress, reduces stress levels, and mitigates pain. Subsequent studies have demonstrated that such contact correlates with lower cortisol levels, underscoring the physiological significance of caregiver-infant proximity (Feldman et al., 2010). With advances in understanding the parvocellular oxytocin system and its interaction with cutaneous sensory input, it has become evident that the calming and soothing effects of holding and hugging probably may involve pathways that trigger oxytocin release in response to tactile stimulation. The oxytocin released within the brain subsequently serves to decrease anxiety, reduce stress and pain, and promote a state of calmness.

## 4 Clinical practices that involve physical closeness and oxytocin release

### 4.1 Skin-to-skin contact after birth

Mothers and their babies are often put in skin-to-skin contact immediately after birth. The practice of skin-to-skin contact substitutes for some of the effects offered by an innate holding behavior, whereby a mother who has given birth brings the newborn to her chest, to hold it, warm it, feed it and to protect it. During skin-to-skin contact maternal chest skin temperature starts to fluctuate and temperature increases. Skin to skin contact also triggers maternal oxytocin release (Matthiesen et al., 2001; Nissen et al., 1995), which is in part associated with milk-ejection but also with increased skin temperature due to vasodilation in the chest area (Bystrova et al., 2007). In addition, the chest is provided with a special type of cutaneous nerves, which travel together with the vagal nerve to the NTS and from there on to the oxytocinergic fibers of the PVN and SON (Eriksson et al., 1996). By these special anatomical and physiological characteristics, the skin overlying the chest becomes particularly well equipped for giving and receiving sensory information involving touch, light pressure and warmth.

When placed on the mother's chest the newborn initiates an innate breast crawling behavior, whereby it reaches the breast and starts suckling, representing an innate social approach behavior (Widström and Matthiesen AS, 1989). The mother being in skin-to-skin contact also engages in more social interaction with her child (Bystrova et al., 2009). These social effects in mother and baby might correspond to the oxytocin linked approach behavior induced by gentle touch.

At the same time both the mother and the baby become calmer, their levels of pain, anxiety and cortisol are reduced. In the newborn peripheral cutaneous temperature rises, a sign of decreased sympathetic tone. Additionally, signs of enhanced vagal/parasympathetic/nerve activity are induced. The latter effects are consistent with increased milk production in the mother and enhanced growth in the newborn (Bystrova et al., 2007), and such effects align with the oxytocin effects induced by cutaneous fibers that respond to firmer types of stimulation.

In addition, sensory stimulation of the skin may activate shorter, spinal nerve reflexes that modify the effects. For example, the skin of the chest is provided with cutaneous sensory neurons influencing autonomic pathways involved in insulin/glucose regulation. Activation of such nerves could explain why infant blood glucose levels rise during skin-to-skin contact (Christensson et al., 1995a,b).

#### 4.1.1 Kangaroo treatment

Kangaroo care or treatment involves repeated skin-skin contact between parents and preterm infants. Kangaroo treatment involves more or less continuous stimulation of cutaneous nerves for days to weeks and has been linked to substantial increase in the rate of growth and development of the child, better milk production and better bonding between mother and infant (Wang et al., 2021; White-Traut et al., 2013; Uvnäs Moberg, 2024).

## 5 Consequences of lack of physical contact

Physical contact plays a significant role throughout an individual's lifespan. The manner in which individuals experience physical contact varies across different stages of life, as do the potential consequences associated with its absence.

### 5.1 Birth and separation

When a newborn baby is separated from the mother it begins to cry. However, if it is put into skin-to-skin contact, the crying stops immediately. Babies who remain separated continue to scream until they are put in skin-to-skin contact. This simple procedure demonstrates the extremely calming effect of close contact with the mother immediately after birth presumably involving oxytocin linked effects (Christensson et al., 1995a,b).

### 5.2 Failure to thrive

Findings show that young children in maternity homes who lack adequate social and physical interaction often develop “failure to thrive syndrome.” One contributing factor is often insufficient stimulation of cutaneous nerves through physical contact, which may lead to inadequate activation of the oxytocinergic system. The failure to thrive syndrome encompasses not only impaired development of social skills but also a compromised immune system and delayed physical growth. As previously described, activation of the oxytocin system plays a critical role in the development of social skills. Additionally, it facilitates vagally mediated stimulation of the endocrine system within the gastrointestinal tract, which supports digestive functions, anabolic processes, and overall growth. To achieve optimal functioning during childhood, digestive and anabolic processes require additional sensory stimulation through suckling or physical contact. Infants who are tube fed often do not gain weight adequately unless they receive supplementary sensory input, such as increased touch or the use of a pacifier for non-nutritive sucking (Uvnäs-Moberg and Widström, 1987). During the first years of life, growth hormone is not yet the primary regulator of growth, so the endocrine activity of the gastrointestinal tract and endocrine pancreas—particularly insulin—play a more significant role in stimulating growth (Benyi and Sävendahl, 2017; Uvnäs-Moberg and Widström, 1987).

### 5.3 Depression, anxiety, and cardiovascular disease

In adults, a distinct pattern of effects is observed in response to insufficient tactile and social interaction. Loneliness—particularly prevalent among older individuals and widespread during the COVID-19 pandemic—is linked to increased rates of depression, anxiety, and serves as a risk factor for cardiovascular disease (Holt-Lunstad, 2021; Jain and Dhall, 2025). These outcomes may be attributed to inadequate direct stimulation of cutaneous afferents, as well as to psychological factors such as the absence of familiar individuals, which may be associated with oxytocin release from parvocellular neurons in the PVN under normal conditions.

### 5.4 Manual therapies

Manual therapies have been utilized across diverse cultures for centuries to enhance wellbeing and manage a variety of medical conditions, with numerous positive outcomes documented. These modalities encompass a broad spectrum of approaches, ranging from classical Swedish massage, which targets muscle tissue, to gentler techniques that primarily stimulate the skin. Manual therapy may involve light or firmer touch and can include stroking motions or static pressure alone. Additionally, both the frequency and duration of treatments are subject to variation depending on the specific regimen employed.

We propose that oxytocin is one of the key mechanisms activated by massage and tactile stimulation. Such stimulation may activate oxytocin release from parvocellular oxytocinergic neurons in the PVN, which in turn influence the levels of social interaction, fear, pain, stress, wellbeing, growth, etc. Indeed, clinical studies demonstrate that manual therapies, massage in particular, can induce effects that represent different aspects of the oxytocinergic effect spectrum. Below a few studies indicating activation of oxytocinergic mechanism in response to manual therapies will be presented.

Massage-like therapy has been shown to give rise to increased social interaction. Even attachment and bonding between mothers and their infants can be reinforced (Field, 2016). Cortisol levels and blood pressure were lowered by massage, and in addition the levels of anxiety decreased (Field, 2019). Children in day care, who received a short period of massage daily became calmer and less aggressive and more cooperative in addition to experiencing less headache and stomach-ache as reported by both staff and parents, than children in a control group (Von Knorring et al., 2008). Massage may have beneficial effects in the treatment of mood disorders, e.g., symptoms of depression are reduced by manual treatment (Eggart et al., 2019).

Massage therapy for premature infants has been demonstrated to enhance weight gain compared to those who did not receive such treatment (Field, 2019). This growth-promoting effect is especially evident in young infants. Additionally, fetal development may be positively influenced when mothers undergo massage during pregnancy; these mothers exhibited reduced stress and gave birth to infants with higher birth weights than those born to mothers who did not receive massage (Glover et al., 2002).

However, only a few studies have reported increased oxytocin levels in response to manual therapies in humans. One such example is the study by Li et al. (2019), which showed that foot and hand massage were associated with elevated oxytocin levels. The limited number of studies demonstrating increases in circulating oxytocin is not unexpected, as most of the oxytocin-mediated effects of massage are likely driven by central release within the brain rather than by changes detectable in peripheral blood.

## 6 Discussion

The findings presented in this review underscore the essential role of touch, physical proximity, and other forms of tactile interaction in human development and overall health. A major focus of this article is to highlight the importance of the oxytocinergic system, particularly the parvocellular neurons in the PVN, for the effects mediated through activation of cutaneous sensory afferents. The association between tactile stimulation and the oxytocin system has a broad significance for emotional regulation, stress reduction, and the prevention of major health issues throughout the lifespan. Oxytocin is not a solitary player, the oxytocinergic system is linked to and influenced by several other more established neurotransmitter systems such as serotonergic, noradrenergic, dopaminergic, cholinergic, opioidergic and many other neuronal pathways. We propose that the oxytocinergic system is of fundamental importance as a basic regulatory system favoring growth and reproduction.

A growing body of evidence supports the efficacy of touch-based interventions not only in addressing developmental challenges but also for mitigating psychological and physiological disorders in adults. The type of stimulation of the skin matters. Both the release pattern of oxytocin in plasma, and the oxytocin linked effect patterns may vary depending on the location and intensity of the stimulation. The effects observed following activation of cutaneous afferents also vary according to the age of the individual receiving the treatment. Growth-promoting effects tend to be more prominent in younger individuals, while stress-relieving and antidepressant effects are more commonly observed in older individuals (McGlone et al., 2024).

Social interactive behaviors may involve subsequent phases of distinct oxytocin linked effect patterns. For example, gentle touch may activate oxytocin neurons which initiate interaction in the receiving individual and decrease anxiety and induce wellbeing. In a subsequent phase firmer, static touch, e.g., in response to holding, may stimulate a different pool of oxytocin neurons which then may be linked to more intense antistress and growth promoting effects, which encompasses the calm and connection response.

The oxytocin linked effects induced during social interaction, will over time be developed into conditioned reflexes and induced by the presence of the person(s) normally triggering it, in early life most often the mother. Data even suggest that those individuals who receive more closeness in infancy and early life develop secure attachment which may correspond to a stronger expression of the activity in the oxytocinergic system, or the calm and connection system. It can of course not be excluded that such long-term effects also involve physiological or epigenetic changes influenced by oxytocin release. Recently oxytocin has been shown to stimulate enzymes (TET 2) involved in demethylation of the oxytocin genes, an important mechanism by which the activity of oxytocin neurons and possibly their receptors may be reinforced (Maejima et al., 2025).

The continuous occurrence of social contact later during life is linked to advantages in health such as reduced occurrence of cardiovascular disease, which in the light of the reasoning above could represent consequences of the repeated tactile stimulation (or of conditioned effects) on the oxytocinergic system. The activity of the oxytocinergic system is reinforced by central sensory cues that mediate the sensation/experience of warmth, support, familiarity and safety. A recent study showed that administration of oxytocin into the brain together with physical touch increased the rate of wound healing, emphasizing how central oxytocin may reinforce the effect of oxytocin released by somatosensory stimulation (Schneider et al., 2025).

### 6.1 Oxytocin and stress

Basically, oxytocin released in response to all types of non-noxious stimulation dampens stress responses, including the activity of the HPA axis, inflammation, as well as of the sympathetic nervous system. However, stress may also inhibit oxytocin levels (Morgan et al., 2024). This reciprocal relationship may help explain why stress can produce physiological and behavioral outcomes that are opposite to those induced by oxytocin-linked touch and social interaction.

### 6.2 Safety and familiarity

The oxytocinergic effects elicited by sensory stimulation can be inhibited under conditions perceived as unsafe. Research has demonstrated that the presence of an unfamiliar individual can suppress oxytocin release during milking in cows and inhibit milk ejection in women. Similar inhibitory effects extend to social interactions (Newton and Newton, 1967; Bruckmaier et al., 1993). This inhibition may result from the subconscious perception of a stranger or unfamiliar person as a potential risk or source of insecurity.

During certain forms of stress or challenging situations, both the oxytocinergic system and the stress system may be activated simultaneously. Under these circumstances, the oxytocinergic system may function as a buffering mechanism, mitigating the adverse effects associated with conditions such as illness and trauma (Takahashi, 2021).

The fight-or-flight response and the oxytocin-driven calm and connection system are both believed to be ancient from an evolutionary perspective. When the oxytocinergic system is active, the body directs energy toward healing, growth, and reproduction. In fact, a decrease in the oxytocin response during certain stressful situations may actually help protect the calm and connection system, as it can only function well when there is a sense of safety and tranquility.

Finally, the discovery of cutaneous afferents that may respond differently to various types of tactile stimulation—and thereby may produce distinct physiological and behavioral effects—opens up the possibility for more targeted tactile therapies. For example, gentle touch techniques may be better suited for encouraging social interaction, enhancing wellbeing, and reducing anxiety, while firmer tactile stimulation is likely more effective for addressing pain, stress-related symptoms, or promoting growth.

Although the present review highlights the theoretical potential for designing tactile interventions with more targeted effects, it is important to emphasize that controlled clinical evidence remains limited. Nevertheless, several recent studies provide preliminary support for the clinical relevance of affective touch and CT-fiber stimulation. For example, affective maternal touch has been shown to enhance physiological synchrony and bonding processes in preterm mother–infant dyads (Grochowska et al., 2024), and CT-optimal dynamic touch reduces physiological arousal in preterm infants (Manzotti et al., 2019). Moreover, a recent systematic review reports alterations in CT-fiber processing across several psychiatric conditions and discusses the therapeutic potential of affective touch-based interventions (Papi et al., 2025). These findings suggest that tactile stimulation may hold promise as a complementary approach in clinical contexts, although further controlled trials are required to establish efficacy, optimal parameters, and long-term outcomes.

### 6.3 Conclusion

Advancing our knowledge of the physiological functions of cutaneous afferents enables a clearer explanation for the significant and beneficial impacts of social interaction and physical closeness on health. Additionally, by distinguishing the effects of different types of touch and their associated neural processes, more targeted and effective tactile interventions can be developed to support health and wellbeing across the lifespan.
